# Prospective association between tobacco smoking and death by suicide: a competing risks hazard analysis in a large twin cohort with 35-year follow-up

**DOI:** 10.1017/S0033291717000587

**Published:** 2017-04-12

**Authors:** A. E. Evins, T. Korhonen, T. H. Kinnunen, J. Kaprio

**Affiliations:** 1 Massachusetts General Hospital, Boston, MA, USA; 2 Harvard Medical School, Boston, MA, USA; 3 Department of Public Health, University of Helsinki, Helsinki, Finland; 4 Department of Public Health and Clinical Nutrition, University of Eastern Finland, Kuopio, Finland; 5 National Institute for Health and Welfare, Helsinki, Finland; 6 Behavioral Science Consulting, Hopkinton, MA, USA; 7 Institute for Molecular Medicine (FIMM), University of Helsinki, Helsinki, Finland

**Keywords:** Nicotine dependence, prospective cohort studies, smoking, suicide, tobacco

## Abstract

**Background:**

The relationship between smoking and suicide remains controversial.

**Method:**

A total of 16 282 twin pairs born before 1958 in Finland and alive in 1974 were queried with detailed health and smoking questionnaires in 1975 and 1981, with response rates of 89% and 84%. Smoking status and dose, marital, employment, and socio-economic status, and indicators of psychiatric and somatic illness were assessed at both time points. Emergent psychiatric and medical illness and vital status, including suicide determined by forensic autopsy, were evaluated over 35-year follow-up through government registries. The association between smoking and suicide was determined in competing risks hazard models. In twin pairs discordant for smoking and suicide, the prospective association between smoking and suicide was determined using a matched case–control design.

**Results:**

Smokers had a higher cumulative suicide incidence than former or never smokers. Heavy smokers had significantly higher suicide risk [hazard ratio (HR) 3.47, 95% confidence interval (CI) 2.31–5.22] than light smokers (HR 2.30, 95% CI 1.61–3.23) (*p* = 0.017). Compared with never smokers, smokers, but not former smokers, had increased suicide risk (HR 2.56, 95% CI 1.43–4.59), adjusting for depressive symptoms, alcohol and sedative–hypnotic use, and excluding those who developed serious somatic or psychiatric illness. In twin pairs discordant for smoking and suicide, suicide was more likely in smokers [odds ratio (OR) 6.0, 95% CI 2.06–23.8].

**Conclusions:**

Adults who smoked tobacco were more likely to die by suicide, with a large, dose-dependent effect. This effect remained after consideration of many known predictors of suicide and shared familial effects, consistent with the hypothesis that exposure to tobacco smoke increases the risk of suicide.

## Introduction

Tobacco smoking has been associated with suicide in a number of studies (Hemenway *et al.* 1993; Tverdal *et al.* 1993; Doll *et al.* 1994; Miller *et al.* 2000*a*; Tanskanen *et al.* 2000; Iwasaki *et al.* 2005; Lucas *et al.* 2013; Schneider *et al.* 2014), but because smokers have higher prevalence of chronic somatic, psychiatric and addictive disorders that confer independent risk for suicide (Grant *et al.* 2004; Hasin *et al.* 2005; Hughes, 2008; Lawrence *et al.* 2009), interpretation of this association remains highly controversial. Some studies have reported that the association between tobacco smoking and completed suicide is independent of potential confounds such as co-morbid psychiatric illness (e.g. depression) and excessive alcohol use (Tanskanen *et al.* 2000; Iwasaki *et al.* 2005; Lucas *et al.* 2013; Schneider *et al.* 2014), while others report that the association is due to heavy alcohol use and psychiatric co-morbidity (Hemmingsson & Kriebel, 2003). Consensus on the nature of the association is needed and could influence suicide risk assessment and broaden our understanding of the harms of tobacco smoking. To reduce the public health burden of suicide mortality, prospective and longitudinal research is needed to improve prediction and identify modifiable risk factors for suicide (Franklin *et al.* 2017). To investigate whether tobacco smoking is independently and possibly etiologically associated with suicide, we evaluated the prospective association between tobacco smoking and change in smoking behavior with subsequent suicide over 35 years in a large, population-based twin cohort, controlling for multiple confounders.

## Method

The Finnish Twin Cohort (Kaprio & Koskenvuo, 2002) includes 16 282 twin pairs alive and aged at least 18 years in 1975. The cohort is made up of 4184 monozygotic (MZ) and 9257 dizygotic (DZ) pairs, with 2841 of unconfirmed zygosity due to non-response or inconsistency in response to zygosity queries, is 49.4% female and had an average age of 35.1 years in 1974. Cohort members received extensive health questionnaires in 1975 and 1981. Response rates were 89% and 84%, respectively.

### Smoking status categories

Smoking was categorized as ‘never’ (fewer than 100 cigarettes lifetime), ‘former’ or ‘active’ smokers in 1975. Further, if smoking status was unchanged at the 1981 assessment, participants were classified as ‘persistent never smokers’, ‘persistent former smokers’ or ‘persistent active smokers’. Participants who were active smokers in 1975 and former smokers in 1981 were classified ‘quitters’; those who were non-smokers in 1975 and smokers in 1981 were ‘initiators’. The category of ‘active’ smokers included both daily and non-daily smokers. Daily smokers reported number of cigarettes smoked per day (cpd) at each time point, and those who smoked >20 cpd were considered heavy smokers.

Detailed information on potential confounders of the relationship between smoking and suicide was collected by questionnaire in 1975 and 1981 and through government registry query for the 35-year follow-up period. Questionnaires included information on well-established predictors for suicide such as marital, employment and socio-economic status, physician-diagnosed somatic illness, depressiveness, and alcohol and sedative–hypnotic medication use.

Respondents completed the four-item (range 4–20) Life Satisfaction Scale (LSS) in 1975 and 1981 to assess pre-existing depressiveness as a proxy for depressive illness. They were asked to rate on a five-point Likert scale interest in life (1 = very interesting, 5 = very boring), happiness (1 = very happy, 5 = very sad), ease of living (1 = very easy, 5 = very hard) and feeling of loneliness (1 = not at all lonely, 5 = very lonely). LSS scores have been linearly correlated with concurrently assessed Beck Depression Inventory (BDI) ratings in a general population sample (*r* = 0.60), and were found to be highly predictive of development of depressive illness in a 15-year prospective longitudinal study in a healthy community sample [dissatisfied (LSS 12–20) *v.* satisfied (LSS 4–6); odds ratio (OR) 6.7, 95% confidence interval (CI) 4.2–10.9] (Koivumaa-Honkanen *et al.* 2004*a*, *b*). LSS scores have demonstrated significant association with completed suicide over a 20-year follow-up, independent of age, sex, alcohol use, smoking status and physical activity (Koivumaa-Honkanen *et al.* 2001). In a mixed clinical sample, LSS scores were associated with anxiety, depressive and psychotic disorder diagnoses, and with use of antidepressant and sedative–hypnotic medications, alcohol and drug use, and BDI scores (Koivumaa-Honkanen *et al.* 1996).

Sedative–hypnotic use was self-rated in 1975 and 1981 on a five-point scale and used in the analysis as no *v.* any use. Excess alcohol use was self-rated at these time points, defined as >42 g/day for men and >28 g/day for women, and binge drinking defined as >1 alcohol-related blackout in the prior year (Carlsson *et al.* 2003).

Psychiatric and somatic co-morbidity was assessed with national registry queries. Receipt of antipsychotic medication at any time from 1975 to 2004 and receipt of antidepressant medication from 1995 to 2004 was identified through the Social Insurance Institution (SII) of Finland (Cannon *et al.* 1998). Antidepressant use was used as an indicator of depressive illness because the psychiatric diagnosis for which an antidepressant was prescribed was not systematically recorded until 2000. Eligibility for psychiatric disability pension at any time from 1975 to 2004 was identified through Finnish pension registers (Harkonmaki *et al.* 2008). Twins with smoking-related somatic illnesses that increase suicide risk, such as cancer, diabetes, cardiovascular and pulmonary diseases such as emphysema, asthma and chronic bronchitis, were identified using self-report and Finnish medical registers to 2011. People with psychiatric or somatic illness were removed from the sample to create a healthy subcohort without tobacco-related somatic conditions for some sensitivity analyses (see Tables 3 and 4).

### Mortality

Information on vital status was obtained from the Population Register Center, which tracks all vital events in Finland. Dates of death and emigration were obtained to 2011. Causes of death were obtained from Statistics Finland. Suicide cases were identified from International Classification of Diseases rubrics. Forensic autopsy by specialist forensic pathologists with support from forensic chemical analyses is mandatory in Finland for sudden and unexpected deaths. Thus the diagnosis of suicide on official death certificates in Finland is considered reliable and valid (Varnik *et al.* 2012).

### Data analyses and statistical methods

Standard statistical methods were used for descriptive analyses, taking into account cohort sampling based on twin pairs. As twins within a pair are not statistically independent observations, robust estimates of standard errors were obtained for individual-based analyses (Williams, 2000).

We first evaluated suicide rates by smoking status using a competing risk model. Because smoking causes diseases that may cause a cohort member to die before she/he might have died by suicide, we used competing-risks regression models in the survival data analysis instead of Cox regression to investigate the effect of smoking status on risk of death by suicide (Lau *et al.* 2009; Haller *et al.* 2013). Mortality not due to suicide was defined as the competing risk and we modeled the subdistribution hazard using Stata's stcrreg procedure. We tested the assumption of proportionality behind competing-risk regression by a covariate × time interaction. Data were censored by date of emigration or end of follow-up (31 December 2011). We then evaluated whether this association remained after excluding persons with major psychiatric or somatic illness. In models excluding persons with major psychiatric or somatic illness, we then evaluated the association between smoking behavior and suicide in an age-adjusted model controlling for factors that may be independently related to suicide. We planned to retain age, sex, LSS scores, sedative–hypnotic medication use, excess daily alcohol use and binge alcohol use in final models. Other covariates were retained if significantly associated with suicide.

To further explore whether the association between smoking and suicide remained after controlling for genetic and familial suicide risk factors, we investigated the relationship of smoking status with suicide in twin pairs discordant for both suicide and active smoking. Using a matched case–control design, we tested ORs using McNemar's test, and then we ran a pairwise survival model. Analyses were run in Stata/SE 12.1 (USA) on 9 July 2013.

## Results

There were 313 suicides in the cohort between 1976 and 2011. Among 26 020 respondents with known smoking status in 1975, there were 232 suicides (see Table 1). Forensic autopsy was conducted in 25% of all deaths in the cohort and in 98% (228/232) of suicide deaths. The overall mortality of the twin cohort members did not differ from that of the Finnish population (Kaprio, 2013).

**Table 1.** Characteristics of cohort members with smoking status in 1975 by vital status in 2011

	Alive	Non-suicide deaths	Suicides	All
Total *N*	17 876	7912	232	26 020
Smoking status, *n* (%)				
Never smoker in 1975	8752 (49.0)	3649 (46.1)	51 (22.0)	12 452 (47.9)
Former smoker in 1975	2846 (15.9)	1400 (17.7)	35 (15.1)	4281 (16.5)
Active smoker in 1975	6278 (35.1)	2863 (36.2)	146 (62.9)	9287 (35.7)
Sex, *n* (%)				
Men	8217 (46)	4297 (54)	182 (78.5)	12 696 (49)
Women	9659 (54)	3615 (46)	50 (21.5)	13 324 (51)
Mean age at baseline, years (s.d.)				
Men	29.8 (8.5)	46.9 (14.2)	33.3 (12.3)	35.6 (13.5)
Women	30.6 (9.6)	54.0 (14.4)	30.0 (9.9)	36.3 (15.2)
Marital status, % married				
Men	52.5	70.1	45.1	58.4
Women	55.3	52.9	52	54.7
Binge drinking, %				
Men	40.3	39.0	61.3	40.2
Women	10.4	4.7	22.0	8.9
Mean life satisfaction score (s.d.)				
Men	8.5 (2.8)	9.0 (3.2)	10.0 (3.2)	8.8 (2.9)
Women	8.5 (2.9)	9.2 (3.0)	9.9 (3.7)	8.7 (3.0)
Psychiatric disability pension by 2004, %				
Men	3.5	6.5	8.7	4.6
Women	4.9	5.9	21.7	5.2
State reimbursable antipsychotic medication, %				
Men	2.1	3.2	4.4	2.5
Women	2.7	4.4	26.0	3.2

s.d., Standard deviation.

### Prospective association between tobacco smoking in 1975 and completed suicide to 2011

**Fig. 1.**
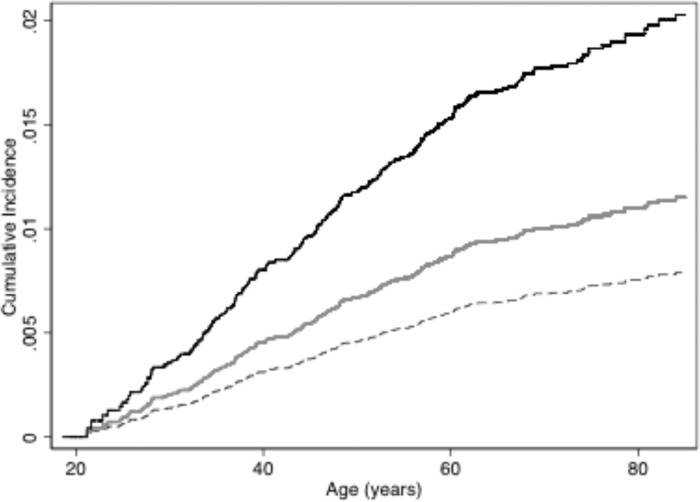
Cumulative incidence of suicide by smoking status. Cumulative incidence of suicide by smoking status in 1975 estimated from a competing risk survival model adjusted for sex and age. The upper curve (––) represents active smokers in 1975, the middle curve (––) represents former smokers in 1975 and the bottom curve (---)represents never smokers in 1975. Based on 26 020 persons in the cohort in 1975 and 232 suicides to 2011.

Those who reported being active smokers in 1975 had a higher risk of suicide from 1976 to 2011 than those who reported never smoking [hazard ratio (HR), 2.59, 95% CI 1.87–3.62], with little variation by sex or age (Table 2). The proportionality assumption was not violated (time × smoking interaction *p* = 0.35, time × sex interaction *p* = 0.46). In addition, risk for suicide was elevated in those who were baseline (1975) smokers but with follow-up initiated from 1 January 1986 with follow-up to 2011 (1986–2011, 160 suicides, HR 2.89, 1.94–4.31). Correspondingly, an analysis in cohort members alive as of 1 January 1996 with follow-up until the end of 2011 showed an association with smoking at baseline (1975) (1996–2011, 72 suicides, HR 2.73, 1.52–4.88). Heavy smokers in 1975 had higher suicide risk over 35 years of follow-up (HR 3.47, 95% CI 2.31–5.22) than daily smokers who smoked less (HR 2.3, 95% CI 1.61–3.23) (*p* = 0.017, test of homogeneity of regression coefficients), with never smokers as overall reference (HR 1). Age of smoking initiation among active smokers, i.e. initiation before age 18 years compared with initiating later, was not associated with suicide risk (data not shown). Both non-daily and daily smokers had elevated risk of subsequent suicide over never smokers that did not differ significantly from each other when modeled separately (HR 2.44, 95% CI 1.29–4.62, and HR 2.60, 95% CI 1.87–3.62, respectively) (not shown in Table 2). Active smokers had a higher cumulative suicide incidence than never smokers at every age (except the oldest age group where the HR point estimate is high but the sample size is small, limiting power to detect an effect) (Table 2, Fig. 1). Active male smokers had the highest suicide risk (70/100 000 person-years), and never-smoking women the lowest (7.3/100 000 person-years). Those who had quit smoking did not have elevated suicide risk, though HR point estimates were greater than unity (Table 2).

**Table 2.** Risk among former and active smokers in 1975 compared with never smokers for suicide in the Finnish Twin Cohort 1976–2011 under a competing risk model^a^

Variables	All	Men	Women	18–30 years	30–59 years	⩾60 years
Never smokers: reference^b^	1.00	1.00	1.00	1.00	1.00	1.00
Former smokers	1.46 (0.95–2.25)	1.46 (0.9–2.39)	1.38 (0.52–3.71)	1.38 (0.72–2.64)	1.42 (0.77–2.63)	1.17 (0.16–8.64)
Active smokers	2.59 (1.86–3.59)	2.52 (1.71–3.72)	2.83 (1.57–5.11)	2.75 (1.77–4.27)	2.34 (1.40–3.93)	3.23 (0.58–17.8)
Suicides, *n*	232	182	50	122	102	8
Total *N*	26 020	12 696	13 324	11 322	12 417	2281

Data are given as hazard ratio (95% confidence interval).

a Estimates for all subjects were adjusted for sex and by three age groups (each also adjusted for sex).

b Never smokers reported smoking fewer than 100 cigarettes in their lifetime.

### Prospective association of tobacco smoking in 1975 on suicide risk to 2011, excluding those with co-morbid psychiatric and medical conditions

The risk of suicide to 2011 remained elevated among active smokers in 1975 relative to never smokers when those who qualified for state reimbursable antipsychotic medication (1964–2004) or psychiatric disability pension (1975–2004), or both, 34 of 232 suicides (14.6%), were excluded from the cohort (HR 2.77, 95% CI 1.93–3.99) (*n* = 198 suicides). When those who reported excess alcohol use in 1975 (⩾42 g/day for men and ⩾28 g/day for women) were removed, 4% of the sample, *n* = 800 (7%) men and *n* = 187 (2%) women, the risk of suicide remained elevated among active smokers to 2011 (HR 2.56, 95% CI 1.62–4.04). The risk of suicide also remained elevated among active smokers (HR 2.30, 95% CI 1.43–3.68) after excluding those broadly defined as ill, aged over 60 years, or at high risk of somatic disease prior to 1983 (Kujala *et al.* 2002) (*n* = 123 suicides) (Table 3). Excluding those with incident malignancy to 2011 according to the Finnish Cancer Registry (*n* = 4500, with 2739 deaths, 15 suicides), the risk of suicide among smokers remained elevated in the remaining healthy cohort of 21 520 persons (HR 2.50, 95% CI 1.80–3.49).

**Table 3.** Risk among former and active smokers, compared with never smokers in 1975, for suicide in 1976–2011 under a competing risk model, excluding persons with known psychiatric or somatic illness^a^

Variable	No antipsychotic medication^b^	No mental health disability pension^c^	No mental health disability pension or antipsychotic medication	No excess alcohol use by self-report in 1981^d^	No mental health disability pension, antipsychotic medication or excess alcohol use	No known tobacco-related somatic illness^e^
Never smokers: reference	1.00	1.00	1.00	1.00	1.00	1.00
Former smokers	1.46 (0.93–2.30)	1.46 (0.92–2.33)	1.50 (0.93–2.41)	1.70 (0.96–3.01)	1.92 (0.94–3.94)	1.61 (0.89–2.91)
Active smokers	2.65 (1.88–3.75)	2.72 (1.92–3.85)	2.77 (1.93–3.99)	2.56 (1.62–4.04)	2.84 (1.61–5.02)	2.30 (1.43–3.68)
Suicides, *n*	211	209	198	94	62	123
Total *N*	25 267	24 952	24 562	18 424	14 256	10 306

Data are given as hazard ratio (95% confidence interval).

a Smoking status (never, former, active) for these analyses is from the 1975 survey.

b Model excluding those who used antipsychotic medications between 1964 and 2004.

c Model excluding those who had a disability pension due to any mental health problem between 1976 and 2004.

d Model excluding those who reported using on average ⩾42 g of alcohol/day (men, *n* = 800) or ⩾28 g of alcohol/day (women, *n* = 187).

e Model excluding persons broadly defined as ill or at high risk of tobacco-related disease prior to 1983, not at work (housework and full-time students not excluded), and aged over 60 years in 1982 (Kujala *et al.* 2002).

### Multivariable analyses

To evaluate the impact of additional potential confounding factors, in cohorts that did and did not exclude those with co-morbid psychiatric and medical conditions as defined above, we ran models with smoking status in 1975, sex and one additional variable to see whether inclusion of the variable influenced the association between smoking status in 1975 and suicide to 2011. We considered a decrease of 5% or more in the HR for suicide (HR 2.59 for active smokers, see Table 2) to be sufficiently large for the variable to be retained for additional analyses. When added to the model, neither socio-economic variables nor employment status substantially affected the association of smoking on suicide.

LSS score, excess daily alcohol use, binge alcohol use, and sedative–hypnotic medication use in 1975 modestly attenuated the association of active smoking with subsequent suicide (HRs 2.23–2.44) and were retained in the model.

In sex-adjusted analysis, LSS score in 1975 was strongly associated with elevated suicide risk (HR 1.14 per unit LSS score increase, 95% CI 1.10–1.18). In a categorical analysis, when those in the dissatisfied and very dissatisfied categories were compared with the satisfied category, suicide risk was elevated (HRs 2.51 and 4.25, respectively).

Excess alcohol use in 1975 was also associated with elevated suicide risk over the 35-year follow-up period in univariate analyses assessed as sex-specific excess alcohol use (⩾42 g/day for men and ⩾28 g/day for women) (HR 2.02, 95% CI 1.05–3.92), and as binge drinking, defined as more than one alcohol-associated blackout in the prior year (HR 2.29, 95% CI 1.43–3.64) (see Table 4).

**Table 4.** Risk of death by suicide from 1982 to 2011 by stability of smoking behavior from 1975 to 1981^a^

	Multivariate model HR (95% CI)			
Variables^b^	All	Men	Women	Age–sex adjusted only without other covariates or mental health exclusions
Persistent never smokers: reference	1.00	1.00	1.00	1.00
Initiators	1.42 (0.56–3.61)	1.78 (0.65–4.93)	0.00 (0.00–0.00)^c^	2.31 (1.14–4.70)
Persistent former smokers	1.66 (0.76–3.64)	1.48 (0.58–3.80)	2.96 (0.78–11.2)	1.56 (0.83–2.95)
Quitters	2.13 (0.99–4.60)	2.21 (0.89–5.45)	1.97 (0.41–9.56)	2.28 (1.23–4.22)
Persistent active smokers	2.56 (1.43–4.59)	2.63 (1.30–5.35)	2.35 (0.82–6.76)	3.27 (2.11–5.08)
Use of sedative–hypnotic medication	2.10 (1.17–3.80)	2.37 (1.23–4.55)	1.40 (0.39–5.00)	
Excess alcohol use	1.57 (0.80–3.06)	1.77 (0.90–3.51)	0.00 (0.00–0.00)^c^	
Binge drinking	2.18 (1.34–3.53)	2.07 (1.25–3.43)	4.30 (1.20–15.32)	
Life satisfaction scale score in 1975	1.06 (0.99–1.14)	1.08 (1.01–1.16)	0.97 (0.78–1.21)	
Life satisfaction scale score in 1981	1.05 (0.99–1.12)	1.02 (0.95–1.09)	1.19 (1.07–1.34)	
Suicides, *n*	102	82	20	153
Total *N*	15 231	7773	7458	21 304

HR, Hazard ratio; CI, confidence interval.

a The first column shows a fully adjusted model that includes mental health-relevant covariates and sex and excludes persons with antipsychotic medication use and disability pension due to mental disorders (see Method for details). Results are presented for men and women together in the first column, and then adjusting for covariates separately for men and women in the second and third columns. The age–sex-adjusted model without covariates or exclusions is shown in the last column for comparison.

b Persistent never smokers were non-smokers in 1975 and 1981. Initiators were non-smokers in 1975 and smokers in 1981. Persistent former smokers were former smokers in 1975 and 1981. Quitters were smokers in 1975 and non-smokers in 1981. Persistent active smokers were smokers in both 1975 and 1981. Data for use of sedative–hypnotic medication, excess alcohol use and binge drinking were from the 1975 assessment. Life satisfaction is entered as a continuous variable, range 4–20, with higher scores indicating greater dissatisfaction.

c The HRs are based on zero cases after excluding those with psychiatric register records.

### Effect of change in smoking behavior between 1975 and 1981 and risk for subsequent suicide

Persistent active smokers (smokers at both time points) had the highest risk for suicide. Those who initiated smoking between the surveys had an increased suicide risk compared with those who were stable non-smokers at both time points (Table 4). Smokers who quit between 1975 and 1981 had a lower point estimate for suicide risk (HR 2.28) compared with persistent smokers (HR 3.27), but the number of suicides was only 11, so power was low for this comparison (*p* = 0.19).

The HR for suicide in active smokers in 1975 and 1981 was reduced from 3.27 to 2.90 and 2.85, respectively, in models containing problem alcohol use variables but remained highly significant. In the multivariable analysis of smoking and these potential confounders, the HR for suicide in active smokers relative to never smokers dropped to 2.14 (95% CI 1.33–3.45) in the model with all cohort members (*n* = 138 cases from 1981 onwards in 1810 persons). In this model, LSS in 1975 and 1981 and binge drinking were also independently associated with suicide. In a model with the same covariates that excluded persons with active or future use of antipsychotic medications or disability pension due to psychiatric illness, the HR for active smokers was 2.56 (95% CI 1.43–4.59) (Table 4).

### Effect of smoking on subsequent suicide risk among those who received no prescriptions for antidepressant medications

Antidepressant prescription data were available from 1995 to 2004, and recording of diagnosis for which antidepressant medication was prescribed began after the year 2000. Thus we ran analyses of the subcohort with no antidepressant medication prescriptions over that 10-year period as a sensitivity analysis in a sample enriched for those without affective disorders, e.g. depression diagnosed by a physician and treated. In a competing risk model in the subcohort of 22 584 persons alive on 1 January 1995 with smoking data and 76 suicides between 1995 and 2011, compared with never smokers, active smokers had a HR of 2.91 (95% CI 1.63–5.17) for suicide with no adjustments or exclusions other than age or sex. In this subcohort, 18 470 persons received no antidepressant prescriptions, 1676 received prescriptions for antidepressant medications for 3–9 months, and 2438 received prescriptions for antidepressant medications for more than 9 months. Among never users of antidepressants, there were 40 suicides, nine among never smokers, seven among former smokers, and 24 among active smokers (in 1975); among them, the risk of suicide was 2.50 (95% CI 1.15–5.42) for active smokers. If we further excluded those with antipsychotic use or disability pension for psychiatric illness, the HR for active smokers among those who received no prescriptions for antidepressant medication was 3.26 (95% CI 1.32–8.08). Further adjustment for pre-existing depressiveness (LSS score) in 1975, binge alcohol use in 1975, or sedative–hypnotic medication use did not significantly change the risk (HR 3.12, 95% CI 1.20–8.13).

Assessed by smoking change between 1975 and 1981, the risk estimate (HR) for suicide 1995–2011 among those who did not receive antidepressant medication (1995–2004) was 4.85 for persistent active smokers (*p* = 0.017), 7.20 (*p* = 0.006) for those who initiated smoking between 1975 and 1981, and 2.78 for those who quit smoking (*p* = 0.23) compared with consistent never smokers. This model excluded those with antipsychotic use or disability pension for psychiatric illness, and adjusted for pre-existing depressiveness (LSS score 1975 and 1981), binge alcohol use 1975 and 1981, and self-reported sedative–hypnotic medication use in 1975 (data not shown).

### Within-pair analysis of smoking and suicide

To further control for shared genetic and environmental risks for suicide, we evaluated twin pairs doubly discordant for smoking and suicide by ascertaining twin pairs in which one twin died by suicide whereas the co-twin was alive in 2011 or had died from another cause, in which smoking status was known for both co-twins in 1975, and in which one twin was an active smoker in 1975 while the co-twin was a never smoker. In all, 28 twin pairs were doubly discordant (eight MZ, 18 DZ, two uncertain zygosity). Of these, the twin who committed suicide was an active smoker in 24 pairs (seven MZ, 15 DZ, two uncertain zygosity), and the never-smoking twin was alive or died from another cause, while the converse was true for four pairs [overall OR 6 (24/4), 95% CI 2.1–23.8; OR 7 for MZ pairs, McNemar *p* = 0.034; OR 5 for DZ pairs, *p* < 0.005]. A test for homogeneity of ORs across zygosity groups indicated no difference by zygosity (*p* = 0.80). Likewise, the risk estimates did not differ by sex (men OR 5.67, women OR 7.00, *p* = 0.86).

Among the doubly discordant pairs, the co-twin was alive at the end of follow-up in 22 pairs, while in five pairs, the co-twin had died from natural causes. Only one pair (MZ) was such that the co-twin, who was an occasional smoker, had death from accidental poisoning (not considered suicide), while the suicide case was the never smoker. We ran a pairwise survival model in which the hazard for the smoking twins was compared with the hazard of the non-smoking co-twin, and the HR was 5.5 (95% CI 1.90–16.0, *p* = 0.002), with HRs of 7.0 and 4.3 for MZ and DZ pairs, respectively.

## Discussion

In this large, population-based sample, active tobacco smoking was dose-dependently associated with increased risk of suicide, while former smoking was not, in a temporal sequence consistent with a causal relationship. This association held for all age groups, except the oldest, which was underpowered. The association remained after excluding those with reliably assessed antipsychotic and antidepressant medication use, psychiatric disability and tobacco-related somatic illness, including cancer. It also remained after adjusting for pre-existing depressiveness as a proxy for depressive illness, sedative–hypnotic use and excess alcohol use. Finally, in the first, to our knowledge, analysis among twin pairs who were doubly discordant for smoking and suicide, as a control for unmeasured genetic and environmental confounding, smoking was also strongly associated with suicide. Thus, this report extends findings from prior reports conducted from cohorts with shorter follow-up periods and fewer sources from which to control for confounders (Tanskanen *et al.* 2000; Iwasaki *et al.* 2005; Li *et al.* 2012; Lucas *et al.* 2013; Schneider *et al.* 2014) and implicates tobacco smoking as an environmental risk factor for suicide, indicating added risk for suicide independent of prior psychiatric and medical diagnosis or treatment.

Strengths of the study are the highly reliable, autopsy-based suicide determination, rigorous controls for potential confounding factors with complete, registry-based psychiatric and medical illness diagnosis and treatment information, the long follow-up period for suicide outcomes that increases confidence that the observed association is not due to pre-existing conditions that increase the risk of suicide, e.g. psychiatric co-morbidity hypothesis, or to the fact that tobacco smoking causes painful and debilitating diseases such as cancer and increases risk for suicide through that mechanism, e.g. the medical co-morbidity hypothesis, and the ability to conduct a within-twin-pair analysis of the association.

The results support the hypothesis that chronic exposure to tobacco smoke is an additional risk factor for suicide that is ameliorated by smoking cessation or reduced in those who are able to quit (Miller *et al.* 2000*b*; Hughes, 2008; Covey *et al.* 2012). A causal biological relationship between tobacco smoking and suicide is plausible. Neurocognitive and neurobiological evidence implicate chronic tobacco use in structural and functional abnormalities in brain reward networks implicated in suicide (Durazzo *et al.* 2010; van Heeringen & Mann, 2014). Nicotine exposure alters synaptic plasticity throughout the striatum, amygdala and hippocampus, a process that reversibly affects neurobiological processes broadly (Levine *et al.* 2011; Huang *et al.* 2013; Kandel & Kandel, 2014).

Abnormalities in multiple systems are observed in both smoking and suicide, including serotonergic and receptor-linked signaling pathways (Pandey, 2013), inflammatory mediators (Rom *et al.* 2013), and neurotrophins and neurotrophin receptors. Brain-derived neurotrophic factor (BDNF) is a much studied member of the neurotrophin family, and genetic variation in the BDNF gene is strongly associated with smoking initiation (Liu *et al.* 2010; Thorgeirsson *et al.* 2010; Tobacco Genetics Consortium, 2010; Breetvelt *et al.* 2012). Further, smoking quantity, severity of nicotine dependence and smoking cessation affect BDNF expression levels (Bhang *et al*. 2010; Jamal *et al*. 2015; Zhang *et al*. 2016). Thus, BDNF expression in the brain is regulated by neurotransmitter systems involved in nicotine use.

Chronic nicotine exposure has direct downstream effects on dopaminergic and glutamatergic neurotransmitter systems with roles in impulsivity and decision-making (Durazzo *et al.* 2016) that are implicated in the etiology of suicide (Kirch *et al.* 1987; Oquendo *et al.* 2014) and which could have both independent effects and additive effects with other static and acute suicide risk factors, consistent with these data. Impulsivity increases with nicotine exposure, normalizes with abstinence, and recurs with re-challenge after abstinence (Kayir *et al.* 2014; Kolokotroni *et al.* 2014). In psychiatric samples, smokers have greater impulsivity, aggression, suicidal ideation and suicide attempts and, among those with depression, lower indices of serotonin function (Malone *et al.* 2003). Nicotine exposure moderates the effect of dopamine receptor gene polymorphisms on major depressive disorder incidence (Korhonen *et al.* 2014). Additionally, tobacco smoke contains multiple other compounds which may contribute, whose effect on central nervous system function in humans is poorly known (US Department of Health and Human Services, 2014). Finally, hypoxia has been postulated as the underlying mechanism for the association between both living at high altitude and suicide (Kim *et al.* 2011) and smoking and suicide (Aubin *et al.* 2011).

Whereas a strength of this study is its strong control for potential confounders, the primary limitation is the possibility of incomplete ascertainment of potentially confounding factors related to both smoking and suicide. We assessed contributions of indicators of diagnosed and undiagnosed psychiatric morbidity at two time points with the LSS as a proxy for pre-existing depressiveness, excess daily and binge alcohol use, sedative–hypnotic use, unemployment, marital status, and other demographic characteristics. We cannot rule out entirely the possibility that these factors changed differentially in smokers *v*. non-smokers over the 35-year follow-up period; however, data from subsets of the cohort presented in this paper were available on questionnaire-based measures between 1975/1981 and 2011. Analyses of differential stability of these risk factors from 1975/1981 to 2011 in smokers *v*. never smokers did not reveal substantial change over time in these self-report measures by smoking status. We additionally assessed contribution of and removed from analyses those with psychiatric disability and antipsychotic and antidepressant medication use over the 35-year follow-up. For this purpose, we considered treatment for psychiatric illness as an indicator for diagnosed psychiatric illness. We were not able to evaluate the impact of treatment for psychiatric illness on suicide rates. We acknowledge that it could be assumed that treatment for psychiatric illness would reduce risk for suicide. Some well-established risk factors such as depression status and trauma history, prior self-injurious thoughts and behavior in self, family members or friends and acquaintances were not explicitly assessed. The LSS questionnaire to assess hopelessness, stress and isolation, all previously identified as risk factors for suicide, was used as a proxy for pre-existing depressiveness, and those receiving antidepressant treatment were identified. We did not consider illicit drug use, which, in this cohort, was rare (Agrawal *et al.* 2008). As expected, suicide was strongly associated with these indicators of psychiatric illness in this sample, and adjustment for these factors attenuated but did not abolish the relationship between smoking and subsequent suicide risk over 35 years. The association between smoking and suicide observed in this study may be elevated by psychiatric illness not captured, but it is not plausible that an unexplained psychiatric variable associated with tobacco smoking could be responsible entirely for a dose-related, 2.5-fold increase in suicide rates. Residual confounding due to imperfect information on the known and measured confounders would need to be more substantial than is plausible to account for the observed strong association, given our use of multiple sources of data. The observed effect was so persistent that to negate the finding, an unanticipated confounder would have to have both a very strong effect and a very biased distribution within the sample, making the likelihood of such a confounder highly implausible (Kotz *et al.* 2015; Leone & Schnoll, 2015). Further, in the within-pair analysis controlling for unmeasured genetic and environmental factors, in 24 of the 28 twin pairs discordant for both smoking and suicide, the twin that died from suicide was the smoker. An experimental design in which some smokers were assigned to become abstinent would be required to understand whether smoking is a causal risk factor. In this study, smoking cessation was associated with reduced risk. However, we cannot rule out whether person-level characteristics associated with successfully quitting smoking are also associated with reduced risk for suicide. The findings are concordant with reports that public policies that reduce smoking on a population level are associated with reduced suicide rates in locations where they are implemented (Grucza *et al.* 2014), supporting a causal relationship, but it is possible that there are societal factors in these geographic areas that reduce suicide risk.

These results provide evidence of a strong, statistically independent, dose-related association between smoking and completed suicide from a large, well-controlled, population-representative cohort with the longest follow-up to date and the first to report a within-twin-pair analysis of smoking and suicide. They support and extend prior reports that active but not former smoking is associated with suicide (Iwasaki *et al.* 2005; Li *et al.* 2012), with heavy smoking associated with greater suicide risk than lighter smoking (Iwasaki *et al.* 2005). They suggest that tobacco smoking is an environmental factor that is possibly etiologically associated with suicide. The findings are intriguing in light of a recent meta-analysis reporting smoking abstinence, compared with continued smoking, associated with reduced depression, anxiety, and stress and increased positive mood and quality of life, with effect sizes comparable with antidepressant treatment (Taylor *et al.* 2014). The results support the hypothesis that exposure to tobacco smoke is a risk for suicide and that reduced risk of suicide is yet another health benefit of smoking cessation. Study of chronic neurobiological effects of tobacco smoke constituents may aid the effort to understand the biological underpinnings of suicide. Further study of the clinical utility of adding smoking status to suicide screening efforts is needed, as is further study of the interaction between smoking and other known static and acute risk factors for suicide, e.g. access to means and life stressors, to build better predictive models of suicide risk (Franklin *et al*. 2017).

## Data Sharing Statement

The data cannot be shared for two reasons. The twin cohort, though representative of the population at large, is a special population. If a person is known to be from Finland, to be a twin and the birth date and sex are known, this uniquely identifies two-thirds of the twin pairs in our cohort, and the remainder with high likelihood of recognition. This is because only a few twin pairs are born on any single day in the country. The survival analyses require birth date, date of entry into the analysis, and possible death date to provide accurate estimates. Second, we access register-based morbidity information, and we have permission to conduct analyses using the register data only at the University of Helsinki. Interested scientists are welcome to request additional analyses by email or by personal visit to Helsinki.
